# Oral Health-Related Quality of Life (OHRQoL) of Children with Down Syndrome and Their Families: A Cross-Sectional Study

**DOI:** 10.3390/children8110954

**Published:** 2021-10-22

**Authors:** AlBandary Hassan AlJameel, Huda AlKawari

**Affiliations:** 1 Department of Periodontics and Community Dentistry, College of Dentistry, King Saud University, Riyadh 11451, Saudi Arabia; 2 Department of Pediatric Dentistry and Orthodontics, College of Dentistry, King Saud University, Riyadh 11451, Saudi Arabia; hkawari@ksu.edu.sa

**Keywords:** oral health-related quality of life, children, family, Down syndrome, Saudi Arabia

## Abstract

As individuals with Down syndrome often suffer from oro-facial abnormalities which can affect their oral health as well as their and their family’s quality of life, this link was examined in the present study. Using a descriptive cross-sectional design, 63 parents of children with Down syndrome who attended two special daycare centres in Riyadh, Saudi Arabia, were surveyed using a self-administered validated questionnaire. The findings yielded by the Statistical Package for the Social Sciences (SPSS Inc., Chicago, IL, USA) version 17 revealed that in 34.9% children and 46% of their families, quality of life was affected by oral health. Moreover, 54% children experienced physical pain, which was severe in 22.2% of the cases. Further analyses revealed that families’ emotional lives were negatively affected by children’s oral health status. Therefore, as oral health in children with Down syndrome exerts significant adverse impacts on different aspects of their lives and those of their families, timely provision of required oral health care is warranted.

## 1. Introduction

The risk of developing oral diseases is higher among individuals with disabilities (including Down syndrome) relative to those who do not have any form of impairment or disability [1,2]. As people with Down syndrome are also more prone to suffer from oro-facial conditions such as malocclusion, periodontal disease, and soft tissue disturbances (i.e., inverted lips and protruding tongue) [3,4,5,6], it is imperative to study the oral health status and its consequences in this population.

Although no association between poor oral health and mortality has been established, it adversely affects morbidity and might exacerbate the existing diseases and conditions [7], thus increasing the burden on individuals and governments [7]. Although the link between poor oral health and individuals’ wellbeing and quality of life (QoL) [8,9] is well established, limited research focusing specifically on individuals with Down syndrome exists [10,11,12,13]. The available evidence, however scant, points to negative impacts of oral health status on the quality of life in this population. Consequently, the Oral Health-Related Quality of Life (OHRQoL) instrument developed specifically for children and adolescents with Down syndrome by AlJameel and her colleagues [11] requires further validation. Therefore, this study aimed to assess the OHRQoL for children with Down syndrome and their families using the validated OHRQoL tool.

## 2. Materials and Methods

### 2.1. Study Design

This descriptive cross-sectional study was conducted from June 2020 to May 2021 and included children with Down syndrome that attended two daycare centres in Riyadh, Saudi Arabia (SAUT and DSCA).

### 2.2. Target Population/Sample Size

The data for this study was obtained by surveying the parents of 63 children with Down syndrome aged 10−14 years that attended the aforementioned daycare centres. All parents were provided the information sheets explaining the study aims and the nature of their involvement, and were asked to sign a written consent form before completing the questionnaire.

### 2.3. Inclusion and Exclusion Criteria

All children with Down syndrome who attended the aforementioned daycare centres in Riyadh whose parents provided written consent were eligible for participation.

### 2.4. Data Collection/Data Source/Variables

The self-administered and validated Oral Health-Related Quality of Life for Children with Down Syndrome (OH-QOLADS) questionnaire [14] was used to collect the data, and further demographic data and information related to the children’s general and oral health status was provided by their parents.

### 2.5. Data Collection/Data Source

All collected data were entered into an Excel spreadsheet and each entry was verified for quality and completeness. Then the data were analysed using the Statistical Package for the Social Sciences (SPSS Inc., Chicago, IL, USA) version 17, and the findings were presented in the form of percentages and frequencies, with the alpha level of significance set at 0.05. In addition, Spearman’s Rank Correlation Test, Kruskal–Wallis Test, and Mann–Whitney test were conducted to determine the differences in variables of interest between groups.

### 2.6. Ethical Approval

Prior to commencing the study, approval was obtained from the Institutional Review Board at King Khalid University Hospital (Registration no. E-19-3657). As noted above, the guardians or parents provided their written consent for participation.

## 3. Results

### 3.1. Demographic

As shown in Table 1, 73% of participating children were aged 11−13 years and 95% of the mothers were married. Even though 52% of participating mothers had secondary or higher educational levels, 82% were unemployed, while the remaining 13% and 5% held full-time and part-time jobs, respectively.

### 3.2. General Health

The parents were also requested to provide information regarding the general health and oral of their children by rating the provided statements on a three-point scale comprising of the “poor,” “fair,” and “good” categories. As can be seen from Table 2, 73% and 25.4% of children were deemed by their parents to have good general and oral health, respectively.

### 3.3. Overall Rating of the Influence of Oral Health on the Child’s and Family’s Quality of Life

The parents were also asked to rate the effect that their children’s oral health has on the child’s (and family’s) quality of life and 34.9% (46%) indicated that in general it had no impact, as shown in Table 3.

### 3.4. Child’s OHRQoL

Table 4 presents the responses parents provided when rating the different quality of life aspects related to their child’s oral health. The tabulated results indicate that 54% of children experienced physical pain, which was severe in 22.2% of cases. Moreover, in around 41% of the children, oral health issues affected their eating habits. On the other hand, very few children seemed to be affected at the emotional level (e.g., shamefulness, lack of self-confidence, embarrassment) or socially (e.g., withdrawal from family relations).

### 3.5. Family’s OHRQoL

Table 5 shows responses the parents provided when rating different effects of their child’s oral health on family’s quality of life. Frustration (42.9%) seemed to be the most prevalent issue, followed by worry (39.85%), and self-blaming (38.7%), while only 23.8% of the respondents indicated that family sleeping patterns were affected. However, 22.2% of the surveyed parents stated that their child’s oral health led to arguments within the family.

### 3.6. Correlation between Child’s OHRQoL Scores and Child’s Perceived General Health, Child’s Perceived Oral Health, and the Overall Impact of Child’s Oral Health on His/Her Quality of Life

Spearman’s correlation coefficients (r_s_) were calculated for the correlation between child’s OHRQoL scores and child’s perceived general health, child’s perceived oral health, and the overall impact of child’s oral health on his/her QoL at the α = 0.05 significance level and were interpreted as very weak = 0.01−0.19, weak = 0.20−0.39, moderate = 0.40−0.59, strong = 0.60−0.79, and very strong = 0.8−1.0. As shown in Table 6, the correlation between the overall child’s OHRQoL rating and OHRQoL scores was statistically significant at *p* < 0.001. Although the correlation between OHRQoL scores and child’s perceived general and oral health was not statistically significant, children whose general and/or oral health status was rated by their parents as poor tended to have higher OHRQoL scores, and thus lower QoL.

### 3.7. Correlation between Family’s OHRQoL Scores and Child’s Perceived General Health, Child’s Perceived Oral Health, and the Overall Impact of Child’s Oral Health on His/her Family’s Quality of Life

As shown in Table 7, the correlation between the overall family’s OHRQoL rating and family’s OHRQoL scores was statistically significant at *p* < 0.001.

## 4. Discussion

The aim of the present study was to assess the OHRQoL for both children and their families from parents’ perspectives. The obtained results indicate that oral health issues exert negative effects on quality of life at different levels. It was particularly noteworthy that a large percentage of children experienced pain, which was severe in many cases. As a result, several mothers stated that their children’s oral health impacted them both emotionally and socially, whereby they would withdraw from their friends and family members. These observations are in line with the findings yielded by previous studies indicating that people who face problems in expressing their feelings, such as individuals with intellectual disabilities, can act emotionally in response to pain, often altering their behaviors [15].

The results suggested that most parents reported the general health status for the participating children with Down syndrome to be good, while their oral health status was described mainly as fair, and this was comparable to the findings of a recently published study in Sweden [16]. Although, according to their parents, nearly all children had some oral health-related problems, no specific oral health issue was reported, as is typically the case for children with Down syndrome. Nonetheless, severity of their dental problems and their reactions to these issues are likely to vary. For instance, the pain sensitivity response among children suffering from Down syndrome is different from that noted for the general population, often manifesting as a delay regarding the painful stimulus, even though later in life people with Down syndrome may experience pain in a similar manner to the mainstream population [3,17].

In their recent study, Carrada et al. assessed caregivers’ perceptions of the quality of life of children with Down syndrome [10]. Their findings revealed that presence of dental caries, severe malocclusion, and defined malocclusion had negative impacts on both children’s and their families’ OHRQoL. To shed further light on these observations, AlJameel reviewed the QoL measures employed in extant studies and comparted the findings yielded. The author noted that in general, poor oral health had adverse impact on children with different disabilities and their families, whereby their QoL would improve following dental treatment [18].

Sheiham and colleagues similarly examined the effects of children’s oral health on their family lifestyle [19] and found that family activities were often disrupted by the oral health of children, concurring with the reports provided by the mothers that took part in the present study. Frustration, worry, and self-blaming were most frequently reported emotional problems, while some mothers also noted family conflict and disruptions to sleeping patterns due to child’s oral problems.

As this is the first study in which a specific OHRQoL measure (OH-QOLADS) that was developed and validated for use in children with Down syndrome was employed in Saudi Arabia, some limitations need to be noted when interpreting the finding yielded. Specifically, the sample size was relatively small, but the data collection could not be extended to other daycare centres due to closures imposed by the government to combat the spread of COVID-19 infections. Similarly, as a result of including only children with Down syndrome who were enrolled in two daycare centres in Riyadh, the potential for generalizing these findings beyond this population is limited, as children with Down syndrome in different settings might have different experiences and therefore different OHRQoL outcomes. Consequently, it would be beneficial to conduct additional studies with larger and more diverse samples.

## 5. Conclusions

Based on the findings obtained in this study, it can be concluded that inadequate oral health exerts significant negative impacts on different aspects of children’s lives as well as those of their families. As poor oral health status often results in pain and causes emotional and social issues, these children need to receive appropriate care in a timely manner. As the reported impacts on the child and/or his/her family could be caused by disability and its consequences (for example, social isolation and stigmatization), additional investigations aiming to segregate different factors affecting their QoL are needed.

## Figures and Tables

**Table 1:** Descriptive Statistics for the Demographic Characteristics of the Study Sample (*n* = 63).

Variable	*F* (%)
**Child’s Gender**	
Male	27 (43.0)
Female	36 (57.0)
**Child’s Age (in Years)**	
10	2 (3.0)
11	7 (11.0)
12	25 (40.0)
13	14 (22.0)
14	15 (24.0)
**Mother’s Age**	
34–39	11 (17.0)
40–45	15 (24.0)
46–51	28 (44.0)
52–57	6 (10.0)
≥58	3 (5.0)
**Mother’s Occupational Status**	
Working full time	8 (13.0)
Working part time	3 (5.0)
Not working	52 (82.0)
**Mother’s Marital Status**	
Married	60 (95.0)
Divorced	1 (2.0)
Widowed	2 (3.0)
**Mother’s Educational Level**	
Uneducated	8 (13.0)
Primary	12 (19.0)
Intermediary	10 (16.0)
Secondary	14 (22.0)
University	18 (29.0)
Postgraduate Studies	1 (1.0)

**Table 2:** Descriptive Statistics for Subjective Assessment of Child’s General and Oral Health Status.

Variable	Frequency	Percent (%)
**General Health Status (*n* = 63)**		
Poor	3	4.8
Fair	14	22.2
Good	46	73.0
**Child Diagnosed with Medical conditions (*n* = 63)**		
Yes	54	85.7
No	9	14.3
**Oral Health Status (*n* = 63)**		
Poor	17	27.0
Fair	30	46.7
Good	16	25.4
**Oral Health Problems (*n* = 63)**		
Yes	61	96.8
No	2	3.2

**Table 3:** Descriptive Statistics for the Overall Rating of Oral Health-Related Quality of Life Questions.

Variable	Frequency	Percent (%)
**Overall impact of child’s oral health on his/her life (*n* = 63)**		
No impact	41	65.1
Has an impact	22	34.9
**Overall impact of child’s oral health on his/her family’s life (*n* = 63)**		
No impact	34	54.0
Has an impact	29	46.0

**Table 4:** Distribution of Responses Related to the Influence of Child’s Oral Health on the Quality of His/her Life.

Variable	Prevalence *n* (%)		Severity of the Problem *n* (%)		
	Never Happened	Ever Happened	Simple	Moderate	Severe
**Physical (*n* = 63)**					
Pain	29 (46.0)	34 (54.0)	10 (15.9)	10 (15.9)	14 (22.2)
**Daily routine (*n* = 63)**					
Eating	37 (58.7)	26 (41.3)	13 (20.6)	8 (12.8)	5 (7.9)
Speaking	58 (92.1)	5 (7.9)	1 (1.6)	4 (6.3)	0 (0.0)
Teeth Cleaning	48 (76.2)	15 (23.8)	7 (11.1)	3 (4.8)	5 (7.9)
Sleeping	55 (87.3)	8 (12.7)	2 (3.2)	2 (3.2)	4 (6.3)
School Duties	56 (88.8)	7 (11.2)	2 (3.2)	2 (3.2)	3 (4.8)
Playing	55 (87.3)	8 (12.7)	1 (1.6)	3 (4.8)	4 (6.3)
**Emotional (*n* = 63)**					
Crying	49 (77.8)	14 (22.2)	6 (9.5)	2 (3.2)	6 (9.5)
Stop Laughing	51 (81.0)	12 (19.0)	5 (7.9)	4 (6.3)	3 (4.8)
Quietness	49 (77.8)	14 (22.2)	6 (9.5)	5 (7.9)	3 (4.8)
Shamefulness	59 (93.6)	4 (6.4)	2 (3.2)	1 (1.6)	1 (1.6)
Embarrassment	60 (95.2)	3 (4.8)	2 (3.2)	1 (1.6)	0 (0.0)
Lack of Self-Confidence	61 (96.8)	2 (3.2)	2 (3.2)	0 (0.0)	0 (0.0)
Awareness of Mouth Related Problems	61 (96.8)	2 (3.2)	1 (1.6)	1 (1.6)	0 (0.0)
Anger	55 (87.3)	8 (12.7)	3 (4.8)	4 (6.3)	1 (1.6)
Stubbornness	59 (93.6)	4 (6.4)	3 (4.8)	1 (1.6)	0 (0.0)
**Social (*n* = 63)**					
Withdraws from Family Relations	56 (88.9)	7 (11.1)	1 (1.6)	4 (6.3)	2 (3.2)
Withdraws from Friends	60 (95.2)	3 (4.8)	1 (1.6)	2 (3.2)	0 (0.0)
Excluded by Friends	61 (96.8)	2 (3.2)	1 (1.6)	1 (1.6)	0 (0.0)
Teasing	56 (88.9)	7 (11.1)	4 (6.3)	3 (4.8)	0 (0.0)

**Table 5:** Distribution of Responses Related to the Influence of Child’s Oral Health on the Family’s Quality of Life.

Variable	Prevalence *n* (%)		Severity of the Problem *n* (%)		
	Never Happened	Ever Happened	Simple	Moderate	Severe
**Daily routine (*n* = 63)**					
Cancelling Planned Activity	56 (88.8)	7 (11.2)	3 (4.8)	2 (3.2)	2 (3.2)
Affects Work	61 (96.8)	2 (3.2)	2 (3.2)	0 (0.0)	0 (0.0)
Not Enough Time for Other Family Members	55 (87.3)	8 (12.7)	3 (4.8)	4 (6.3)	1(1.6)
Disturbed Sleep	48 (76.2)	15 (23.8)	3 (4.8)	7 (11.1)	5 (7.9)
**Emotional (*n* = 63)**					
Frustration	36 (57.1)	27 (42.9)	2 (3.2)	11 (17.5)	14 (22.2)
Self-Blaming	38 (61.3)	24 (38.7)	3 (4.8)	4 (6.5)	17 (27.4)
Worry	38 (60.2)	25 (39.8)	3 (4.8)	11 (17.5)	11 (17.5)
Anger	54 (85.6)	9 (14.4)	3 (4.8)	3 (4.8)	3 (4.8)
**Conflict (*n* = 63)**					
Arguing with a Family Member	49 (77.8)	14 (22.2)	5 (7.9)	8 (12.7)	1 (1.6)
Jealousy among Siblings	60 (95.2)	3 (4.8)	1 (1.6)	1 (1.6)	1 (1.6)

**Table 6:** Association between Total Child’s OHRQoL and Perceived Health Indicators.

Variable	Child’s OHRQoL Score > 0, Ever Happened					
	Median	Mean	SD	*p*-Value ^1^	r_s_	*p*-Value ^2^
**Child’s Perceived GH**						
P (*n* = 3)	3.0	13.3	19.7	0.971	0.006	0.985
F (*n* = 10)	3.5	8.5	11.4			
G (*n* = 32)	5.0	7.0	6.5			
**Child’s Perceived OH**						
P (*n* = 13)	10.0	12.0	12.5	0.156	−0.215	0.302
F (*n* = 21)	3.0	5.2	4.2			
G (*n* = 11)	3.0	7.5	8.7			
**Overall Child’s OHRQoL Rating**						
No impact (*n* = 23)	3.0	3.7	3.3	<0.001	0.512	0.001 ^a^
Has an impact (*n* = 22)	9.0	11.9	10.6			

^1^ Spearman’s Rank Correlation Test. ^2^ Kruskal–Wallis Test for difference between groups in child’s OHRQoL Scores. ^a^ Mann–Whitney test (we have two categories for the independent variable).

**Table 7:** Association between Total Family’s OHRQoL and Perceived Health Indicators.

Variable	Family’s OHRQoL Score > 0, Ever Happened					
	Median	Mean	SD	*p*-Value ^1^	r_s_	*p*-Value ^2^
**Child’s Perceived GH**						
P (*n* = 2)	9.5	9.5	7.8	0.317	−0.160	0.596
F (*n* = 7)	9.7	9.7	7.7			
G (*n* = 32)	6.0	6.5	5.0			
**Child’s Perceived OH**						
P (*n* = 13)	9.0	9.7	5.1	0.140	−0.234	**0.044**
F (*n* = 20)	4.0	5.2	4.6			
G (*n* = 8)	6.5	8.1	7.3			
**Overall Family’s OHRQoL Rating**						
No impact (*n* = 16)	2.0	3.4	2.7	<0.001	0.59	<0.001 ^a^
Has an impact (*n* = 25)	9.0	9.6	5.7			

^1^ Spearman’s Rank Correlation Test. ^2^ Kruskal–Wallis Test for difference between groups in family’s OHRQoL scores. ^a^ Mann–Whitney test (we have two categories for the independent variable).

## Data Availability

Not applicable.

## References

[1] Choi J., Doh R.M. (2021). Dental treatment under general anesthesia for patients with severe disabilities. J. Dent. Anesth. Pain Med..

[2] Paulson D.R., Pattanaik S., Chanthavisouk P., John M.T. (2021). Including the patient’s oral health perspective in evidence-based decision-making. Bundesgesundheitsbla.

[3] Fiske J., Shafik H. (2001). Down’s syndrome and oral care. Dent. Update.

[4] Hennequin M., Faulks D., Veyrune J.L., Bourdiol P. (1999). Significance of oral health in persons with Down syndrome: A literature review. Dev. Med. Child. Neurol..

[5] Ghaith B., Al Halabi M., Khamis A.H., Kowash M. (2019). Oral health status among children with Down syndrome in Dubai, United Arab Emirates. J. Int. Soc. Prev. Community Dent..

[6] Asokan S., Muthu M.S. (2008). and Sivakumar, N. Oral findings of Down syndrome children in Chennai city, India. Indian J. Dent. Res..

[7] Kassebaum N.J., Smith A.G., Bernabé E., Fleming T.D., Reynolds A.E., Vos T., Murray C.J.L., Marcenes W., GBD 2015 Oral Health Collaborators (2017). Global, regional, and national prevalence, incidence, and disability-adjusted life years for oral conditions for 195 countries, 1990–2015: A systematic analysis for the global burden of diseases, injuries, and risk factors. J. Dent. Res..

[8] Sheiham A. (2005). Oral health, general health and quality of life. Bull. World Health Organ..

[9] Benyamini Y., Leventhal H., Leventhal E.A. (2004). Self-rated oral health as an independent predictor of self-rated general health, self-esteem and life satisfaction. Soc. Sci. Med..

[10] Carrada C.F., Scalioni F.A.R., Abreu L.G., Ribeiro R.A., Paiva S.M. (2020). Impact of oral conditions of children/adolescents with Down syndrome on their families’ quality of life. Spec. Care Dentist.

[11] AlJameel A.H., Watt R.G., Tsakos G., Daly B. (2020). Down syndrome and oral health: Mothers’ perception on their children’s oral health and its impact. J. Patient-Rep. Outcomes.

[12] Oliveira A.C., Pordeus I.A., Luz C.L., Paiva S.M. (2010). Mothers’ perceptions concerning oral health of children and adolescents with Down syndrome: A qualitative approach. Eur. J. Paediatr. Dent..

[13] Amaral-Loureiro A.C., Oliveira Costa F., Eustáquio da Costa J. (2007). The impact of periodontal disease on the quality of life of individuals with Down syndrome. Downs Syndr Res. Pract..

[14] AlJameel A.H.M. (2016). The Development and Testing of an Oral Health-Related Quality of Life Measure for Children/Adolescents with Down Syndrome (OH-QOLADS). Ph.D. Thesis.

[15] Radovich F., Charich G., Vecchi R. (1991). The evaluation of anxiety and analysis of pain perception in Down’s syndrome patients undergoing dental procedures. Minerva Stomatol..

[16] Stensson M., Norderyd J., Van Riper M., Marks L., Björk M. (2021). Parents’ perceptions of oral health, general health and dental health care for children with Down syndrome in Sweden. Acta Odontol. Scand..

[17] Hennequin M., Morin C., Feine J.S. (2000). Pain expression and stimulus localisation in individuals with Down’s syndrome. Lancet.

[18] AlJameell A.H. (2020). Oral health-related quality of life outcomes for individuals with disabilities: A review. J. Clin. Diagn..

[19] Sheiham A., Steele J.G., Marcenes W., Tsakos G., Finch S., Walls A.W. (2001). Prevalence of impacts of dental and oral disorders and their effects on eating among older people; a national survey in Great Britain. Community Dent. Oral Epidemiol..

